# Minimal shedding of the glycocalyx layer during abdominal hysterectomy

**DOI:** 10.1186/s12871-017-0391-6

**Published:** 2017-08-22

**Authors:** Janis Nemme, Robert G. Hahn, Camilla Krizhanovskii, Stelia Ntika, Olegs Sabelnikovs, Indulis Vanags

**Affiliations:** 1 Department of Anaesthesiology and Intensive Care, Riga Stradins University and Paul Stradins Clinical University Hospital, Riga, Latvia; 2 Research Unit, Södertälje Hospital, 152 86 Södertälje, Sweden

## Abstract

**Background:**

Surgery with and without hypervolaemia may cause shedding (breakdown) of the endothelial glycocalyx layer, but the severity of this problem is unclear.

**Methods:**

In this preliminary report of a larger clinical trial, the plasma and urine concentrations of three biomarkers of glycocalyx shedding (syndecan-1, hyaluronic acid and heparan sulfate) were measured in seven patients before, during, and after open hysterectomy. The fluid therapy consisted of 25 ml/kg (approximately 2 l) of Ringer’s lactate, which was infused over 30 min when the surgery started. The resulting plasma volume expansion at the end of the infusion was estimated from the haemodilution.

**Results:**

The mean plasma concentration of syndecan-1 was 21.7 ng/ml before surgery and averaged 19.7 ng/ml during and after the surgery. The plasma concentration of hyaluronic acid decreased from 38.0 to 27.7 ng/ml (*P* < 0.05), while heparan sulfate increased from 3.4 to 5.5 μg/ml (*P* < 0.05). The urine concentrations of syndecan-1 decreased significantly, while they increased for hyaluronic acid and heparan sulfate. Despite the vigorous fluid load, the urine flow did not exceed 1 ml/min.

**Conclusions:**

No clear evidence was found for shedding of the endothelial glycocalyx layer when 2 l of Ringer’s lactate was infused over 30 min during abdominal hysterectomy. Urine analyses yielded patterns of changes that differed from those in plasma.

**Trial registration:**

ISRCTN81005631. Registered May 17, 2016.

## Background

The glycocalyx, a thin mucous layer of substance on the luminal side of the endothelium, is of increasing interest among anaesthetists. It consists of negatively charged, highly organized mesh of glycoproteins (such as syndecans) with attached linear glycosaminoglycans (hyaluronic acid, heparan sulfate and chondroitin sulfate), that harbours plasma proteins (albumin, antithrombin III, and others). This fine layer maintains a low endothelial permeability to plasma proteins, governs local blood flow, contributes to the initiation of the inflammatory process, and counteracts the formation of microthromboses [1].

The glycocalyx is easily and quickly damaged and even broken down (shed) in response to a variety of stimuli, such as inflammation and hypoxia [2]. Hypervolaemia caused by infusion fluids causes shedding via the release of atrial natriuretic peptides (ANPs) from the heart [3]. Avoidance of hypervolaemia could therefore represent an important approach for the anaesthetist when trying to prevent shedding. However, the degree to which shedding occurs in response to crystalloid fluid loading during surgery is not well established.

In the present report, the concentrations of three shedding biomarkers were measured repeatedly in the plasma and urine before, during, and after surgery that involved administration of a large bolus dose (25 ml/kg over 30 min) of crystalloid fluid. The purpose was to examine if a crystalloid bolus causes shedding in the clinical setting and to compare the plasma and urinary concentrations of the shedding products over time. Abdominal hysterectomy was chosen for the project because this surgery is a reasonably wellstandardized procedure associated with more-than-minimal physiological stress and need for infusion fluid. The hypothesis was that increased concentrations of shedding products do occur, but not to the same degree as is observed with sepsis [4], trauma, [5] or surgery that involves ischaemia [6].

## Methods

Between March and June 2016, seven patients (mean age 47 years, range 42–55; body weight 79 kg, range 54–98) scheduled for abdominal hysterectomy were recruited for the present preliminary report, which is a part of a randomized clinical trial aimed at comparing the glycocalyx shedding caused by different forms of general anaesthesia (propofol and sevoflurane) and its relationship to capillary leakage of albumin and the plasma volume expansion as indicated by haemodilution. The study was approved by the Clinical Research Ethics Committee of Riga Stradins University (Trial registration no. 270116-17 L, decision date: January 27, 2016, Officer in charge: P. Stradins) and was registered at controlled-trials.com as ISRCTN81005631. Two patients refused to participate and were replaced by consecutive ones. All reported patients gave us their written informed consent to participate. Inclusion criteria were ages between 25 and 55 years, no chronic cardiopulmonary or renal diseases requiring daily therapy, an expected operating time < 90 min, and expected blood loss <500 ml.

After an overnight fast, the patients were weighed and transferred to the operating theatre. Midazolam (7.5 mg orally) premedication was given the night before the scheduled operation. General anaesthesia was induced with midazolam (0.03 mg/kg), fentanyl (0.025–0.03 mg/kg) and propofol (1.5 mg/kg). Tracheal intubation was facilitated by atracurium (0.5 mg/kg). A urinary catheter was placed in the bladder and a second large cannula (G14) was inserted in an arm vein. Anaesthesia was maintained with either 1.8–2.2% sevoflurane in the inspired air or propofol 1.5-2.5 mg/kg as an intravenous bolus injection followed by an infusion of propofol 10 mg/kg/h during the first 10 min, then 8–9 mg/kg/h over the next 10 min, and thereafter 6–7 ml/kg/h, adjusting the dose to the clinical signs. Lung ventilation was instituted using low flow anaesthesia with 1.0 l of fresh gas/min and a tidal volume of 5–6 ml/kg, as predicted by the body weight, to maintain the end-tidal CO_2_ at ≈40 mmHg. Monitoring consisted of ECG, pulse oximetry, and non-invasive arterial pressure. Two anaesthetists were involved; one provided the anaesthesia and the other (always J.N.) managed the study.

At the start of surgery, an intravenous infusion of Ringer’s lactate (25 ml/kg) was administered over 30 min. After that infusion, no more fluids (or blood products) were administered, except for the propofol infusion and tiny amounts of fluids necessary for drug dilution and administration. The surgical haemorrhage was estimated visually from the blood collected on swabs and dressings and in suction bottles.

Venous blood was sampled in EDTA tubes. A first sample was withdrawn just before the patient was transferred to the operating theatre. Subsequent samples were taken after general anaesthesia had been induced, but just before the fluid load was initiated, and then 30, 60, and 90 min later. A final sample was taken 2 h after the anaesthesia had been terminated. All patients had an indwelling catheter in the bladder; urine was collected from the catheter output on the same occasions as the blood samples were taken.

The plasma and urinary concentrations of shedding products were measured on all collected samples on a single occasion, after having been stored at -80^o^ C, at the Research Unit at in Södertälje Hospital, Sweden, by one of the co-authors (S.N.). Commercially available ELISA kits were used. Syndecan-1 was evaluated with human CD138/syndecan 1 (Diaclone, France), hyaluronic acid with a hyaluronan immunoassay (R&D Systems, Inc., MN, USA), and heparan sulfate with AMS.E-EL-H2364 (Amsbio, Abingdon, UK) with CV% values of 6.2%, <7.2%, and <10%, respectively. The blood haemoglobin (Hb) concentration was measured on a Coulter HMx 5-diff (Beckman Coulter Inc., Brea, CA). The percentage plasma volume expansion induced by Ringer’s lactate was calculated based on the blood haemoglobin (Hb) concentration taken just before the infusion (time 0) and at the end of infusion (time 30). The equation used was:

$$ \mathrm{Volume}\  \mathrm{expansion}\ \left(\%\right)=100\ \left[\left({\mathrm{Hb}}_0/{\mathrm{Hb}}_{30}\right)\left.-1\right)\right]/\left(1-\mathrm{haematocrit}\right) $$

Data with a normal distribution are presented as the mean (standard deviation, SD). Changes were evaluated using the paired *t* test. Data having a skewed distribution were presented as the median (25th–75th percentiles). Due to frequent occurrence of skewed distributions, changes were evaluated by using the Wilcoxon’s matched-pair test. *P* < 0.05 was considered statistically significant.

The complete clinical trial will finally include 24 patients, which is based on a difference in the rise of the plasma concentration of syndecan-1 of 50% between propofol and sevoflurane anaesthesia (power 80% and *P* < 0.05).

## Results

The amount of Ringer’s lactate infused during the first 30 min of surgery amounted to 1964 ± 387 ml. The haemodilution indicated that the plasma volume had increased by to 37 ± 6% at the end of the infusion of Ringer’s lactate. No other fluid or blood products were given. The blood loss was 164 ± 48 ml and the anaesthesia lasted for 100 ± 10 min. All operations were uneventful.

The haemodynamic data are shown in Table 1, upper section.

**Table 1:** Haemodynamics and the plasma and urinary concentrations of shedding products at baseline and during an abdominal surgery procedure and postoperative care (*N* = 7)

	Before surgery	During surgery and PACU	Statistics
Haemodynamics			
Systolic arterial pressure (mmHg)	144 ± 22	112 ± 11	*P* < 0.021
Diastolic arterial pressure (mmHg)	84 ± 15	68 ± 7	*P* = 0.053
Heart rate (bpm)	92 ± 12	69 ± 7	*P* < 0.003
Shedding products, plasma			
Syndecan-1 (ng/ml)	21.0 ± 3.6	19.7 ± 5.1	*P* = 0.312
Hyaluronic acid (ng/ml)	38.0 ± 6.9	27.7 ± 5.3	*P* < 0.016
Heparan sulfate (μg/ml)	3.4 ± 0.9	5.5 ± 0.8	*P* < 0.001
Shedding products, urine			
Syndecan-1 (ng/ml)	42.9 (36.9–139.8)	24.0 (15.6–46.0)	*P* < 0.028
Hyaluronic acid (ng/ml)	10.8 (8.2–29.0)	35.5 (30.9–37.1)	*P* < 0.043
Heparan sulfate (μg/ml)	5.5 (5.1–5.9)	5.7 (5.3–6.7)	*P* < 0.043

Data are the mean ± SD or median (25th–75th percentiles), as appropriate

*PACU* postoperative care unit

**Fig. 1:**
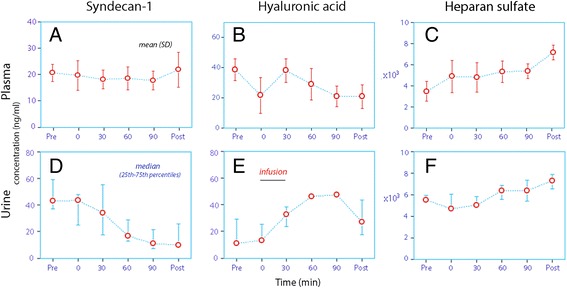
The concentrations of three shedding products of the endothelial glycocalyx layer. Syndecan-1, hyaluronic acid and heparan sulfate were measured both in plasma (**a**-**c** upper row; mean values) and urine (**d**-**f** lower row; median values)

Compared to the baseline values, the plasma concentrations of syndecan-1, which was the key outcome measure, were unchanged during anaesthesia, surgery, and postoperative care (21.0 versus 19.7 ng/ml, respectively; Fig. 1a). There was a minimal increase at 120 min (to 22.4 ng/ml), but a post hoc power analysis showed that 120 patients would be needed to demonstrate statistical significance by *P* < 0.05 with a certainty of 80% for the change in plasma syndecan-1 that occurred between the pre- and postoperative sampling points.

The hyaluronic acid concentrations decreased (from 38.0 to 27.7 ng/ml) while those of heparan sulfate increased (from 3.4 to 5.5 μg/ml; Table 1, middle; Fig. 1b, c).

A plot of the plasma concentrations of these shedding products over time (Fig. 1 top row) illustrates that most of the increase in heparan sulfate occurred before the infusion of fluid and in the postoperative care unit. The sample taken just before initiation of the fluid infusion did not differ significantly from the mean of the samples taken 30, 60, and 90 min later [4.9 ± 1.5 versus 5.2 ± 0.8, respectively; *P* = 0.49].

**Fig. 2:**
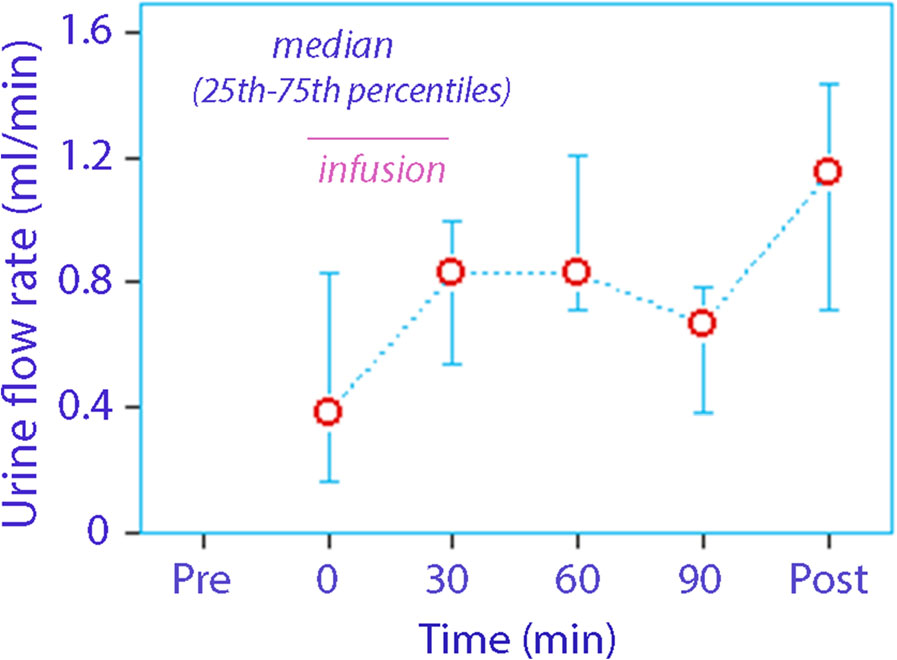
The urine flow rate before, during, and after open abdominal surgery. The flow is typically very low during the surgery despite vigorous volume loading, but increases postoperatively

The urinary concentration of syndecan-1 decreased significantly, while that of hyaluronic acid and of heparan sulfate increased (Table 1, lower; Fig. 1d–f). The average urine flow was maintained below 1 ml/min during the surgical procedures (Fig. 2).

## Discussion

Degradation (shedding) of the glycocalyx layer on the luminal side of the vascular endothelium is an adverse effect of hypervolaemia and surgery. In fact, shedding due to hypervolaemia from volume loading has been a concern for anaesthetists during the past several years [2]. In the present report, three biomarkers commonly used to indicate shedding were monitored, with the overarching aim of studying their relationship to fluid distribution and capillary leakage of albumin during anaesthesia and surgery. However, only minimal changes in the plasma concentrations of these substances occurred during abdominal hysterectomy (Fig. 1). Syndecan-1, which is the most widely used biomarker of shedding, was basically unchanged. Only plasma heparan sulfate levels showed some degree of increase, and mostly before and after the surgery. This glycosaminoglycan plays an important role in the inflammatory process, which is activated by surgery.

The results also demonstrate that the concentrations of shedding products may not show the same pattern in plasma as they do in urine. These findings have relevance to the interpretations of data on shedding products, for which both plasma and urine data have been used. For example, the plasma concentration of syndecan-1 was virtually unchanged, but its concentration in urine decreased markedly during the perioperative period. A comparison between the actual plasma and urine concentrations over time suggests that syndecan-1 was enriched in the urine before the surgery, but not after it. The physiological importance of these differences is uncertain.

The urinary concentrations of hyaluronic acid increased 4-fold, while the plasma concentrations decreased. Here, markedly similar maximum urine levels were obtained for all patients, which might suggest a physiological limit for its excretion. The rise is consistent with previous data suggesting a higher urinary concentration of hyaluronic acid when fluid retention occurs, induced by surgical stress, while no such difference is observed for syndecan-1 [7]. By contrast, the plasma and urine concentrations of heparan sulfate were fairly similar throughout the study, which suggests that this glycosaminoglycan readily equilibrates between the body fluids.

The glycocalyx is known to degrade in response to systemic inflammation, sepsis, ischaemia, trauma, and abdominal surgery [2, 3]. In severe sepsis, high concentrations of syndecan-1 in plasma were defined as being 12 times higher than the baseline level reported in the present study [4]. After trauma, the median plasma syndecan-1 level averaged 3 times higher compared to our baseline, and “high” concentrations averaged 8 times higher [5]. Dramatic elevations, up to 65 times, occurred in response to regional ischaemia during heart surgery in adults [6]. Neonates undergoing heart surgery had a four-fold plasma concentration of syndecan-1 when being weaned after a cardio-pulmonary bypass [8]. In a pig model, the syndecan-1 level in plasma had tripled one hour after sepsis had been induced [9]. In all studies where shedding has been considered to occur, the plasma concentration of syndecan-1 has increased by several multiples. No change of that magnitude was seen in the present work.

The glycocalyx layer is of special relevance to fluid therapy, as shedding increases the vascular permeability for macromolecules [10]. Without this layer, a much shorter intravascular persistence of intravenous fluid can be assumed. Anaesthetists are advised to avoid liberal fluid administration in their patients because hypervolaemia causes the heart to release of ANP, which is a known sheddase [3].

Jacob et al. [11], using an isolated heart model, showed that several natriuretic peptides cause extravasation of fluid. By contrast, convincing evidence for this effect in vivo is scarce. A study using indocyanine green as tracer of blood volume changes reported that 60% of the infused volume of hydroxyethyl starch was extravasated within minutes, which represents an impressive rapid change in fluid distribution that was attributed to hypervolaemia and an assumed ANP-induced shedding of the glycocalyx [12]. This result has gained widespread attention [13], but it might only reflect that the transit time for the dye was overlooked [14].

In a later study that used a similar set-up, Chappell et al. [15] infused patients with 20 ml/kg of hydroxyethyl starch before elective surgery and found an increase in plasma syndecan-1 and hyaluronan of 80%, while the heparan sulfate concentration did not change. Increased concentrations of syndecan-1 were also found in urine. However, the authors had corrected their plasma concentrations for the dilution of serum albumin, which was of the same magnitude. Therefore, the serum concentration may not have increased, and the data could instead reflect the total amount of syndecan-1 and hyaluronan in the circulating blood. Whether this approach reflects shedding would then depend on the volume of distribution of these substances. Shedding might not have occurred if syndecan-1 distributes over a space that is much larger than the plasma volume.

In the present study we recorded a marked haemodilution at the end of infusion, while distribution throughout the extracellular fluid space is known to reduce crystalloid-induced hypervolaemia to only about 15–20% of the infused volume 30 min later [16]. However, no dip in the plasma concentrations of any of the shedding products can be discerned at the end of the infusion as compared to the adjacent data points, which suggests that syndecan-1 diffused quickly from the interstitial fluid to the plasma as soon the latter was diluted with crystalloid fluid (Fig. 1, top row). Similar equilibration across the capillary wall of substances that have a large volume of distribution, such as cystatin C, has previously been reported in association with fluid infusions [17].

A previous study from our group showed no elevation of plasma syndecan-1 in elderly men following infusion of 15 ml/kg of Ringer’s acetate and isotonic saline over 45 min [18]. The present study involved a more rapid infusion of as much as 25 ml/kg of Ringer’s lactate, which supersedes the rate, but not the total volume, of crystalloid fluid that is typically administered during abdominal hysterectomy. This fluid load is expected to have doubled the plasma concentration of ANP [19]. However, no change in plasma syndecan-1 occurred in our patients, suggesting that no clinically relevant shedding occurs in response to fluid loading with buffered Ringer’s solution within the volume range used in common practice. Although ANP sheds the glycocalyx, a doubling of its plasma concentration may not be sufficient for this to occur. Much is known about other agents that cause shedding [20], but none of importance seems to have functioned here except for the presumably surgery-induced inflammation that slightly raised the plasma concentration of heparan sulfate.

We report the urine flow because previous work suggests that different amounts of shedding products are excreted depending on whether the kidneys concentrate the urine [7]. Despite the vigorous fluid load, the urine flow was very low during the anaesthesia, which is a typical finding in the perioperative setting [21]. The flow rates were even quite similar to those found during laparoscopic cholecystectomy where fluid loading was performed [22].

One limitation of this study is that the data represent the first third of a randomized clinical trial that will be presented later in full. However, the consistent lack of elevation of the syndecan-1 concentration, which is the most widely used biomarker used to demonstrate shedding, makes a different result highly unlikely in a larger cohort. The same drugs and doses were used during the induction of anaesthesia, but maintenance was achieved with either propofol of sevoflurane between which a difference in shedding products cannot be evaluated in this preliminary report. Moreover, the ELISA kits used are not specifically designed for urine samples. Two of the manuals mention “biological samples” and the one for hyaluronan is intended for serum and plasma samples. Finally, the concentrations of ANP were not measured.

The long-term goal of the current project study is to quantify capillary leakage of albumin and the crystalloid fluid kinetics in relationship to shedding of the glycocalyx, as well as to examine if the degree of shedding differs depending on whether the anaesthesia is maintained by propofol or sevoflurane. These objectives were not elaborated upon in the present preliminary report, However, the minimal evidence of shedding found in the first seven patients makes it unlikely that shedding would markedly influence capillary leakage and fluid volume kinetics.

## Conclusion

No clear evidence was found for shedding of the glycocalyx layer during an abdominal hysterectomy that incorporated a rapid infusion of 25 ml/kg of Ringer’s lactate. The shedding products showed different time courses in the plasma when compared to the urine.

## Abbreviations

ANP: Atrial natriuretic peptide
ELISA: Enzyme-linked immunosorbent assay
Hb: Blood haemoglobin
PACU: Postoperative care unit
SD: Standard deviation
